# Increased relative wall thickness is a marker of subclinical cardiac target-organ damage in African diabetic patients

**DOI:** 10.5830/CVJA-2012-023

**Published:** 2012-09

**Authors:** Pilly Chillo, Eva Gerdts, Pilly Chillo, Eva Gerdts, Johnson Lwakatare, Janet Lutale

**Affiliations:** Institute of Medicine, University of Bergen, Norway; Institute of Medicine, University of Bergen, Norway; Department of Heart Disease, Haukeland University Hospital, Bergen, Norway; Muhimbili National Hospital and Muhimbili University of Health and Allied Sciences, Dar es Salaam, Tanzania; Department of Heart Disease, Haukeland University Hospital, Bergen, Norway; Muhimbili National Hospital and Muhimbili University of Health and Allied Sciences, Dar es Salaam, Tanzania; Muhimbili National Hospital and Muhimbili University of Health and Allied Sciences, Dar es Salaam, Tanzania

**Keywords:** left ventricular geometry, African diabetes, relative wall thickness

## Abstract

**Objective:**

To assess the prevalence and covariates of abnormal left ventricular (LV) geometry in diabetic outpatients attending Muhimbili National Hospital in Dar es Salaam, Tanzania.

**Methods:**

Echocardiography was performed in 61 type 1 and 123 type 2 diabetes patients. LV hypertrophy was taken as LV mass/height^2.7^ > 49.2 g/m^2.7^ in men and > 46.7 g/m^2.7^ in women. Relative wall thickness (RWT) was calculated as the ratio of LV posterior wall thickness to end-diastolic radius and considered increased if ≥ 0.43. LV geometry was defined from LV mass index and RWT in combination.

**Results:**

The most common abnormal LV geometries were concentric remodelling in type 1 (30%) and concentric hypertrophy in type 2 (36.7%) diabetes patients. Overall, increased RWT was present in 58% of the patients. In multivariate analyses, higher RWT was independently associated with hypertension, longer isovolumic relaxation time, lower stress-corrected midwall shortening and circumferential end-systolic stress, both in type 1 (multiple R^2^ = 0.73) and type 2 diabetes patients (multiple R^2^ = 0.66), both *p* < 0.001. These associations were independent of gender, LV hypertrophy or renal dysfunction.

**Conclusion:**

Increased RWT is common among diabetic sub-Saharan Africans and is associated with hypertension and LV dysfunction.

## Abstract

The co-existence of diabetes with other cardiovascular risk factors, such as hypertension and obesity, may contribute to the association of diabetes with subclinical cardiac target-organ damage such as left ventricular (LV) hypertrophy and dysfunction. In addition, several reports have suggested that diabetes has direct adverse effects on the heart, independent of obstructive coronary artery disease.^1^,^2^ In the Strong Heart study, non-insulin dependent diabetes was associated with a 12 to 14% higher LV mass/height^2.7^ as well as reduced LV systolic function and increased arterial stiffness.^3^ Among hypertensive diabetic African Americans, increased relative wall thickness (RWT) and LV hypertrophy have been found to be more prevalent,^4^,^5^ and earlier development of cardiac end-organ damage than in Caucasians has been suggested.^6^

In sub-Saharan Africa, diabetes and other cardiovascular diseases were considered rare.^7^ As a result, research focus has been on infectious diseases. However, recent publications in the region have shown an increase in the prevalence of diabetes, hypertension and other cardiovascular risk factors,^8^ and a high prevalence of LV hypertrophy, in particular in hypertensive patients, has been reported.^9^ However, there are limited data on subclinical cardiac target-organ damage in diabetic patients.

The aim of the present study was therefore to determine the prevalence and covariates of abnormal LV geometry among type 1 and type 2 diabetes outpatients of African origin attending Muhimbili National Hospital in Dar es Salaam, Tanzania.

## Methods

This study was a prospectively planned follow-up examination of 244 diabetic patients of African origin who participated in a diabetes study programme that included clinical and biochemical examination at Muhimbili National Hospital in Dar es Salaam, Tanzania in 2003–2004.^10^,^11^ Of the total 244 patients who participated in the first survey, 184 patients (75%) were still receiving care at the diabetes outpatient clinic in Muhimbili National Hospital in 2008. Patients were informed about the follow-up study when attending their regular visits at the diabetes outpatient clinic and subsequently invited to participate. All 184 patients agreed to participate and signed informed consent.

A structured questionnaire was used for interviewing the patients on socio-demographic characteristics, history of other cardiovascular risk factors and duration of diabetes. Height and weight were measured and used to calculate body mass index. Waist circumference was measured at the level of the umbilicus and used as a measure of central obesity. Blood pressure was measured using a mercury sphygmomanometer and appropriate cuff size. After five minutes’ rest in the sitting position, a set of three readings was taken five minutes apart. The average of the last two readings was taken as the patient’s clinic blood pressure.^12^ Hypertension was defined as blood pressure ≥ 140/90 mmHg or use of antihypertensive medication.

Fasting capillary blood glucose and glycated haemoglobi (HbA_1c_) levels were measured on spot; blood glucose by a HemoCue AB glucose analyser (Angelholm, Sweden) and HbA_1c_ using a DCA 2000+ analyser (Bayer Inc., New York, USA). Urinary albumin/creatinine ratio (UACR) was measured in a spot morning urine sample using the same DCA 2000+ analyser, which measures urine albumin (in mg/l) and creatinine (in mg/dl) concentrations and calculates the urine albumin-to-creatinine ratio (UACR). Microalbuminuria was defined as UACR > 30 mg/g and macroalbuminuria as UACR > 300 mg/g.^13^ Biochemical tests were performed with the use of a chemistry analyser (Abbott Architect, Illinois, USA) at Muhimbili National Hospital laboratory, which is the National reference laboratory.

All patients gave written informed consent. The study was ethically approved by the Muhimbili University of Health and Allied Sciences’ research and publication committee.

All echocardiograms were performed by the same licensed cardiologist, who had received special training in echocardiography (PC), using a SONOS 7500 Phillips echocardiogram machine. Patients were examined in the left lateral decubitus position using a 3-MHz transducer. The echocardiographic protocol included parasternal long- and short-axis views of the left ventricle, left atrium and aorta, as well as two-, three- and four-chamber images of the left ventricle and pulsed Doppler recordings of LV filling. Spectral tissue Doppler was recorded of mitral annular plane velocity in the apical four-chamber view.

All images were recorded digitally on Magnetic Optical disks, and interpretation of all digital echocardiograms was done at the Department of Heart Diseases, Haukeland University Hospital using a Tomtec (TomTech Imaging Systems GmbH, Unterschielssheim, Germany) work station for post-processing. All studies were first read by the primary investigator and then proof read by the senior investigator, a highly experienced reader (EG).

Quantitative echocardiography was performed following the American Society of Echocardiography guidelines.^14^ LV hypertrophy was considered present when LV mass indexed for height^2.7^ exceeded 49.2 g/m^2.7^ in men and 46.7 g/m^2.7^ in women.^15^ RWT was calculated as the ratio of end-diastolic posterior wall thickness to end-diastolic LV internal radius and considered increased if ≥ 0.43.

Patients were categorised into four LV geometric patterns based on LV mass/height^2.7^ (LVMI) and RWT measurements in combination. Normal geometry was considered present if LVMI and RWT were both normal, concentric remodelling was the combination of normal LVMI and increased RWT, eccentric hypertrophy was the combination of LV hypertrophy and normal RWT, and concentric LV hypertrophy was present if LV hypertrophy and increased RWT were both present.^14^

LV circumferential end-systolic stress (CESS) was estimated at the midwall using a cylindrical model.16 Myocardial contractility was assessed by midwall fractional shortening (MWS), calculated using a previously validated formula, taking into consideration the epicardial migration of the midwall during systole.^17^ Stress-corrected fractional shortening (scFS) and stress-corrected MWS (scMWS) were calculated as the ratio between actual and predicted FS and MWS for actual CESS, respectively, using previously published equations.^17^

Transmitral flow was recorded with pulsed-wave Doppler between the mitral cusp tips in the apical four-chamber view. The early (E) and atrial (A) waves were traced for peak velocities and used to calculate the E/A ratio. Isovolumic relaxation time was measured from the leading edge of the aortic valve closure spike to the leading edge of the mitral valve high-intensity echo in five-chamber view. Early diastolic mitral annular plane velocity (E′) was measured by spectral tissue Doppler in the apical four-chamber view.^18^

## Statistical analysis

Data management and statistical analysis was performed using SPSS for Windows version 18.0. Data are presented as mean ± SD for continuous variables and as percentages for categorical variables. Groups of patients were compared using the χ^2^ test for categorical variables and unpaired Student’s *t*-test, one way ANOVA with Sheffe’s *post hoc* test or general linear model with Sidak’s *post hoc* test for continuous variables, as appropriate. Bivariate correlations were assessed by Pearson’s correlation coefficients. Covariates of increased RWT were identified in the total study population and in groups of type 1 and type 2 diabetes patients by multiple linear regression analyses, run with an enter procedure and co-linearity statistics. A two-tailed *p*-value ≤ 0.05 was considered statistically significant.

## Results

The study population included 61 type 1 and 123 type 2 diabetes patients. Compared to type 1 patients, type 2 patients were older, had longer duration of diabetes and included more hypertensive and obese patients (all *p* < 0.01) (Table 1).

**Table 1:** Demographic And Laboratory Characteristics Of Type 1 And Type 2 Diabetes Patients

*Characteristic*	*Type 1 (n = 61)*	*Type 2 (n = 123)*	p*-value*
Age (years)	21.7 ± 10.6	55.0 ± 9.6	< 0.001
Females, *n* (%)	34 (55)	78 (64)	0.265
Duration of diabetes (years)	8.2 ± 4.5	10.7 ± 6.3	0.005
Body mass index (kg/m^2^)	20.9 ± 4.4	28.4 ± 4.7	< 0.001
Obesity, *n* (%)	2 (3.3)	45 (36.6)	< 0.001
Waist circumference (cm)	74 ± 12	98 ± 11	< 0.001
Systolic blood pressure (mmHg)	117 ± 21	147 ± 22	< 0.001
Diastolic blood pressure (mmHg)	74 ± 14	88 ± 11	< 0.001
Hypertension, *n* (%)	11 (17.7)	100 (82.0)	< 0.001
Pulse pressure (mmHg)	43 ± 12	59 ± 17	< 0.001
Fasting blood glucose (mmol/l)	12.2 ± 4.4	10.4 ± 4.7	0.015
HbA_1c_ (%)	10.9 ± 2.2	9.8 ± 2.3	0.003
Total cholesterol (mmol/l)	4.7 ± 1.6	5.6 ± 1.5	0.001
HDL cholesterol (mmol/l)	1.2 ± 0.4	1.2 ± 0.3	0.855
LDL cholesterol (mmol/l)	3.2 ± 1.3	4.0 ± 1.4	< 0.001
Triglycerides (mmol/l)	1.6 ± 1.6	1.7 ± 1.0	0.617
Serum creatinine (μmol/l)	84 ± 70	106 ± 77	0.058
eGFR (ml/min/1.73 m^2^)	106 ± 47	81 ± 24	< 0.001
Low eGFR, *n* (%)	6 (10)	21 (18)	0.268
Albuminuria, *n* (%)	24 (40.0)	39 (33.6)	0.412
Microalbuminuria, *n* (%)	16 (26.7)	33 (28.4)	0.860
Macroalbuminuria, *n* (%)	8 (13.3)	6 (5.2)	0.077

HbA_1c_ = glycated haemoglobin, HDL = high-density lipoprotein, LDL = low-density lipoprotein, eGFR = estimated glomerular filtration rate.

Compared to type 1 diabetes patients, type 2 patients had larger LV dimensions and higher RWT and LVMI (Table 2). LV systolic chamber function measured as stress-corrected fractional shortening and ejection fraction did not differ between the two groups, while myocardial contractility assessed by stress-corrected midwall shortening was significantly lower among type 2 diabetes patients (Table 2). Measures of diastolic function were also significantly unfavourable in the type 2 diabetes patients (Table 2) However, LV dimension and function did not differ between the two types of diabetes when adjustment for age and systolic blood pressure was done (Table 2).

**Table 2:** Echocardiographic Findings In Type 1 And Type 2 Diabetes Patients

	*Unadjusted*			*Adjusted for age and systolic blood pressure*		
*Echocardiographic finding*	*Type 1 (n = 61)*	*Type 2 (n = 123)*	p-*value*	*Type 1 (n = 61)*	*Type 2 (n = 123)*	p-*value*
Interventricular septum in diastole (cm)	0.91 ± 0.21	1.27 ± 0.31	< 0.001	1.11 ± 0.06	1.16 ± 0.04	0.573
LV posterior wall in diastole (cm)	0.79 ± 0.17	1.06 ± 0.25	< 0.001	0.94 ± 0.05	0.98 ± 0.03	0.622
LV end-diastolic diameter (cm)	4.01 ± 0.63	4.21 ± 0.58	0.036	4.10 ± 0.13	4.16 ± 0.08	0.769
Relative wall thickness	0.40 ± 0.10	0.52 ± 0.19	< 0.001	0.48 ± 0.04	0.48 ± 0.02	0.938
LV mass/height^2.7^ (g/m^2.7^)	33.0 ± 9.6	49.2 ± 16.8	< 0.001	40.6 ± 3.0	45.1 ± 1.8	0.299
Fractional shortening (%)	37 ± 5	35 ± 6	0.176	36 ± 1.3	36 ± 0.8	0.940
Stress-corrected fractional shortening (%)	99 ± 11	99 ± 16	0.942	100 ± 3	99 ± 2	0.739
Ejection fraction (%)	65 ± 7	63 ± 8	0.328	63 ± 2	64 ± 1	0.554
Midwall shortening (%)	16 ± 3	13 ± 3	< 0.001	14 ± 0.7	15 ± 0.4	0.875
Stress-corrected midwall shortening (%)	90 ± 17	74 ± 18	< 0.001	80 ± 3.8	81 ± 2.4	0.918
Transmitral E/A ratio	1.5 ± 0.4	0.9 ± 0.3	< 0.001	1.2 ± 0.8	1.1 ± 0.5	0.226
Deceleration time (ms)	165 ± 52	206 ± 61	< 0.001	191 ± 13	192 ± 8	0.954
Isovolumic relaxation time (ms)	62 ± 16	81 ± 20	< 0.001	78 ± 3.8	73 ± 2.4	0.378
Early tissue Doppler velocity (E′) (cm/s)	10.3 ± 2.3	6.5 ± 2.4	< 0.001	8.3 ± 0.5	7.5 ± 0.3	0.305
E/E′ ratio	9.5 ± 2.4	11.7 ± 4.4	< 0.001	11.2 ± 0.8	10.8 ± 0.5	0.733

**Fig. 1:**
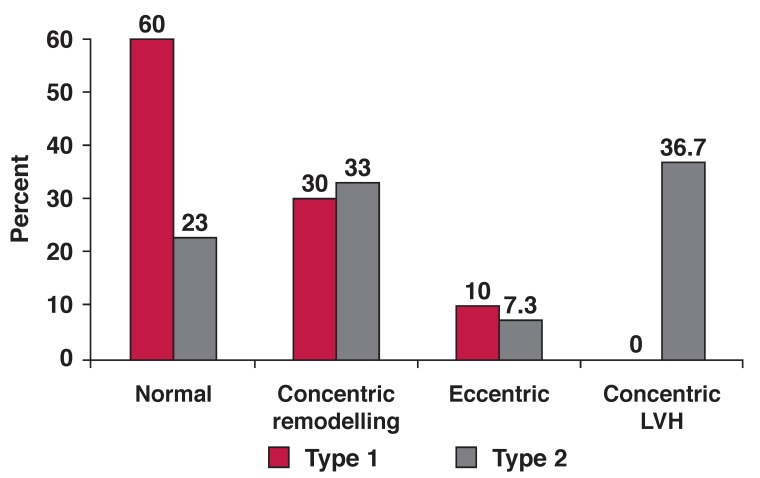
LV geometric patterns in type 1 (red bars) and type 2 (grey bars) diabetes patients. The differences between normal geometry and concentric LVH were statistically significant, both *p* < 0.001.

**Fig. 2:**
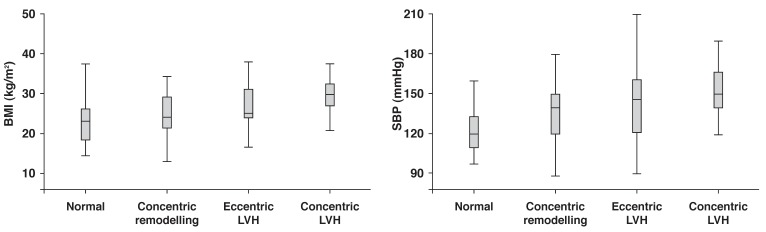
LV geometry in relation to body mass index and systolic blood pressure, and impact on comparison between the different LV geometric patterns; *p* < 0.001 for comparison of body mass index (left panel) and systolic blood pressure (right panel) in the four geometric patterns by ANOVA .

**Fig. 3:**
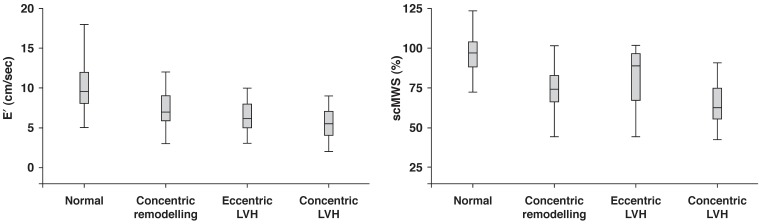
Early tissue Doppler velocity (E′) and stress-corrected midwall shortening (scMWS) in relation to LV geometric patterns; *p* < 0.001 for comparison of E′ (left panel) and scMWS (right panel) in the four geometric patterns by ANOVA .

In the total population, the prevalence of concentric remodelling, eccentric hypertrophy and concentric hypertrophy was 32, 8.3 and 23.7%, respectively. LV geometry differed significantly between type 1 and type 2 diabetes patients as a consequence of more type 2 diabetes patients having concentric LV hypertrophy (Fig. 1). Systolic blood pressure and body mass index were among the most important covariates of LV geometry in the total study population (Figs 2, 3).

In logistic regression analysis involving the total study population, LV hypertrophy (combined eccentric and concentric LV hypertrophy) was associated with obesity, (OR 3.97, 95% CI: 1.65–9.54, *p* = 0.002), hypertension (OR 4.58, 95% CI: 1.32–15.85, *p* = 0.016) and albuminuria (OR 2.31, 95% CI: 1.01–5.27, *p* = 0.047). This was independent of age, gender, type or duration of diabetes (Table 3).

**Table 3:** Independent Predictors Of LV Hypertrophy In The Total Population By Logistic Regression Analysis

*Variable*	*Odds ratio (95% CI)*	*p-value*
Obesity	3.97 (1.65–9.54)	0.002
Hypertension	4.58 (1.32–15.85)	0.016
Albuminuria	2.31 (1.01–5.27)	0.047
Age (years)	1.03 (0.98–1.08)	0.206
Male gender	0.66 (0.28–1.53)	0.329
Type of diabetes (type 1 vs type 2)	0.73 (0.13–4.17)	0.727
Duration of diabetes (years)	0.99 (0.92–1.06)	0.785

The most prevalent types of abnormal LV geometry were concentric remodelling in type 1 diabetes patients and concentric LV hypertrophy in type 2 diabetes patients (Fig. 1). Overall, 58% of the total population had increased RWT. In univariate linear regression analysis, the most important correlates of higher RWT were older age, higher blood pressure and higher log UACR, both in type 1 and type 2 diabetes patients (all *p* < 0.05) (Table 4). In addition, lower eGFR and high-density lipoprotein (HDL) cholesterol were significantly correlated with higher RWT among type 2 but not in type 1 diabetes patients. Having increased RWT was also associated with impaired systolic and diastolic LV function, including lower myocardial contractility, measured as scMWS, and delayed early LV diastolic relaxation, measured as longer IVRT, longer deceleration time and reduced E/A ratio, both in type 1 and type 2 diabetes patients (all *p* < 0.05) (Table 4).

**Table 4:** Correlates Of RWT In The Total Population And In Type 1 And Type 2 Diabetes Patients

	Total population		Type 1		Type 2	
	*r*	p-*value*	*r*	p-*value*	*r*	p-*value*
Age (years)	0.391	< 0.001	0.357	0.005	0.203	0.035
Body mass index (kg/m^2^)	0.237	0.002	0.068	0.605	0.031	0.752
Systolic blood pressure (mmHg)	0.383	< 0.001	0.359	0.004	0.234	0.015
Diastolic blood pressure (mmHg)	0.388	< 0.001	0.331	0.009	0.282	0.003
Fasting blood glucose (mmol/l)	0.029	0.705	0.204	0.118	0.068	0.485
HbA_1c_ (%)	–0.009	0.909	0.113	0.390	0.066	0.496
eGFR (ml/min/1.73 m^2^)	–0.282	< 0.001	–0.076	0.563	–0.319	0.001
HDL cholesterol (mmol/l)	–0.165	0.033	–0.146	0.265	–0.277	0.002
Triglycerides (mmol/l)	0.134	0.082	0.279	0.031	0.079	0.416
Triglyceride-to-HDL cholesterol ratio	0.108	0.163	0.141	0.287	0.175	0.069
Log UACR (mg/g)	0.147	0.059	0.259	0.048	0.194	0.045
E′ (cm/sec)	–0.434	< 0.001	–0.149	0.246	–0.377	< 0.001
LV mass/height^2.7^ (g/m^2.7^)	0.477	< 0.001	0.113	0.389	0.426	< 0.001
E/A ratio	–0.382	< 0.001	–0.321	0.012	–0.241	0.012
Deceleration time (ms)	0.313	< 0.001	0.255	0.047	0.228	0.017
Isovolumic relaxation time (ms)	0.428	< 0.001	0.304	0.017	0.347	< 0.001
Circumferential end-systolic stress (dyne/cm2)	–0.421	< 0.001	–0.349	0.006	–0.557	< 0.001
Midwall shortening (%)	–0.717	< 0.001	–0.619	< 0.001	–0.723	< 0.001
Stress-corrected midwall shortening (%)	–0.755	< 0.001	–0.675	< 0.001	–0.759	< 0.001
E/E′	0.299	< 0.001	–0.158	0.228	0.293	0.002

HbA_1c_ = glycated haemoglobin, eGFR = estimated glomerular filtration rate, HDL = high-density lipoprotein, UACR = urine albumin creatinine ratio.

When multivariate linear regression analyses were performed, higher systolic blood pressure, longer IVRT and low scMWS remained significant covariates of higher RWT both in type 1 and type 2 diabetes patients, irrespective of presence or absence of LV hypertrophy and also adjusted for CESS. In addition, low eGFR continued to be an independent covariate of higher RWT in type 2 diabetes patients. Substituting log UACR for eGFR in the type 1 diabetes patients’ model did not give any independent association either (Table 5).

**Table 5:** Independent Covariates Of Higher RWT In Total Population And In Type 1 And Type 2 Diabetes Patients

	Total population (R^2^ = 0.69*)		Type 1 (R^2^ = 0.73*)		Type 2 (R^2^ = 0.66*)	
*Covariate*	*ß*	p-*value*	*ß*	p-*value*	*ß*	p-*value*
Systolic blood pressure (mmHg)	0.301	< 0.001	0.442	< 0.001	0.233	0.001
Low eGFR (ml/min/1.73 m2)	0.131	0.007	0.009	0.909	0.150	0.024
Low stress-corrected MWS (%)	0.239	< 0.001	0.493	< 0.001	0.156	0.017
Isovolumic relaxation time (ms)	0.170	0.001	0.180	0.041	0.155	0.016
LV mass/height^2.7^	0.187	0.001	0.091	0.284	0.189	0.008
Circumferential end-systolic stress (dyne/cm^2^)	–0.584	< 0.001	–0.682	< 0.001	–0.602	< 0.00
Male gender	0.083	0.065	–0.009	0.905	0.123	0.051

eGFR = estimated glomerular filtration rate, MWS = midwall shortening, *p < 0.001.

In binary logistic regression analysis, including type of diabetes, albuminuria, obesity, history of hypertension and HbA_1c_ level, the independent covariates of increased RWT were: type 2 diabetes (OR 2.7, 95% CI: 1.08–7.00), albuminuria (OR 2.2, 95% CI: 1.01–4.62), obesity (OR 2.6, 95% CI: 1.02–6.58) and hypertension (OR 2.5, 95% CI: 1.02–5.87), all *p* < 0.05.

**Fig. 4:**
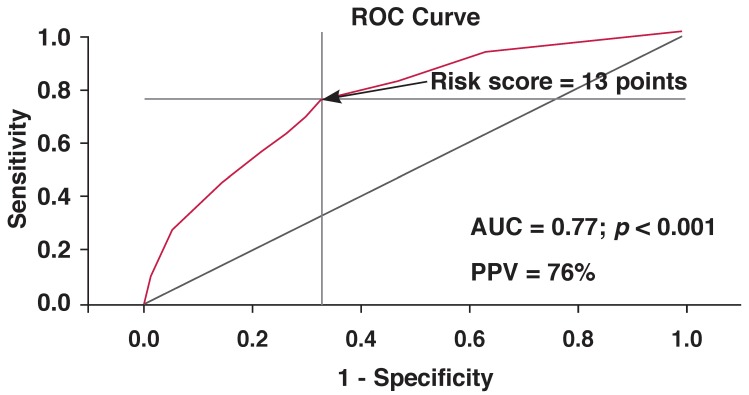
Receiver-operator characteristic (ROC) curve for the clinical risk score with best sensitivity (76%) and specificity (67%) in predicting high relative wall thickness. The cut-off value for the risk score (13 points) identified by the ROC analysis is indicated by an arrow. AUC = area under the curve, PPV = positive predictive value.

A risk score was calculated based on the beta coefficients in this model: risk score = 9*x* (type of diabetes) + 8*x* (albuminuria) + 9*x* (obesity) + 9*x* (hypertension). For each parameter included in the score, a value of 1 was assigned if the variable was present or 0 if it was absent. Therefore the individual risk score varied in this study population between 0 and 35 points. Based on the ROC curve analysis, the optimal cut-off point for the prediction of increased RWT was a score of 13 points (area under the curve = 0.77, *p* < 0.001, sensitivity = 76% and specificity = 67%). This risk score had a positive predictive value of 76% (Fig. 4).

## Discussion

From echocardiographic studies in Caucasians, North American Indians and African Americans, it is well known that diabetes is associated with concentric LV remodelling, and LV hypertrophy is particularly common in patients with combined type 2 diabetes and hypertension.^19^,^20^ However, few studies have reported on LV geometry in diabetic populations from sub-Saharan Africa. Therefore, the present study is among the few to report on prevalence and covariates of abnormal LV geometry in diabetic sub-Saharan African patients.

The study has many interesting findings, adding to current knowledge on diabetic heart disease in Africans, in particular (1) that abnormal LV geometry is common in sub-Saharan African diabetic patients, (2) that concentric remodelling was the most prevalent abnormal LV geometric pattern in this population and associated with reduced LV myocardial contractility and delayed diastolic relaxation, and (3) that a simple algorithm combining everyday clinical and laboratory assessment may be used to identify diabetic patients with high risk of cardiac target-organ damage.

Our findings add to a previous report by Ojji *et al*. on Nigerians with type 2 diabetes.^21^ In their study of 122 patients, abnormal LV geometry was found in 51% of patients, compared to 74% in the present study. Of note, the study by Ojji *et al*.^21^ only included normotensive type 2 diabetes patients, and as demonstrated by our findings, hypertension was a strong covariate of having both LV hypertrophy and increased RWT, probably explaining the higher prevalence of abnormal LV geometry in the present study. As demonstrated, age and systolic blood pressure were the main confounders explaining the difference in LV structure between groups of patients with type 1 or type 2 diabetes.

Hypertension, in particular isolated systolic hypertension, increases in prevalence with aging, mainly as a consequence of arterial stiffening imposing increased load on the left ventricle. Older age has been documented to be particularly associated with increased RWT, and with LV hypertrophy when hypertension coexists.^22^-^24^ However, despite differences in socio-demographic backgrounds, our results were comparable to those reported by Eguchi *et al*. from Japanese hypertensive patients with type 2 diabetes. In their study, including 161 patients, the prevalence of concentric remodelling, eccentric hypertrophy and concentric hypertrophy, respectively, were 29, 16 and 39%.^25^

We found no previous echocardiographic study on LV geometric patterns performed among type 1 diabetes patients from sub-Saharan Africa, and our study is probably the first to describe LV geometry in such patients. As demonstrated by our results, abnormal LV geometry was found in 40% of type 1 diabetes patients. Specifically, 30% of type 1 diabetes patients had concentric remodelling, and this was the most common type of abnormal LV geometry in this group. All six type 1 diabetes patients (10%) with LV hypertrophy had eccentric LV hypertrophy.

Interestingly, none of the type 1 diabetes patients had concentric LV hypertrophy, the most common abnormal LV geometric pattern found among type 2 diabetes patients in the present study. This finding could probably be explained by the low prevalence of hypertension among type 1 diabetes patients in our study (18 vs 82%). Other investigators have reported a higher prevalence of LV hypertrophy among type 1 diabetes patients with nephropathy.^26^

Of note, in the present study population, all type 1 diabetes patients with LV hypertrophy also had albuminuria (results not shown), and albuminuria was identified as a main covariate of LV hypertrophy in multivariate analysis. The beneficial impact of renin–angiotensin inhibition on albuminuria and the prevention of overt renal failure has previously been demonstrated in type 1 diabetes patients with microalbuminuria.^27^ Whether the prevention of progression to overt renal failure with the use of drugs that inhibit the renin–angiotensin system will also prevent progression to LV hypertrophy among type 1 diabetes patients is a question that needs to be answered in future prospective studies in Africans.

The finding that higher RWT was significantly associated with older age and higher blood pressure agree with previous reports from epidemiological studies in North American Indians.^3^ Importantly though, as demonstrated by multivariate analysis in our study, independent associations between increased RWT and measures of systolic and diastolic LV function were found irrespective of presence or absence of LV hypertrophy or hypertension. This is an important finding because it emphasises the need to further stratify patients into the different LV geometric patterns, rather than by presence or absence of LV hypertrophy alone. The finding is particularly important in the African diabetes context, as concentric remodelling (increased RWT with normal LVMI) was found to be the most common abnormal LV geometric pattern in the present study, as also previously reported among African American hypertensive patients.^4^

In 884 children and adolescents with a high prevalence of obesity, Di Bonito *et al*. found that higher triglyceride-to-HDL cholesterol ratio independently predicted higher RWT and concentric LV hypertrophy.^28^ In our study, lower serum HDL cholesterol levels, but not triglyceride-to-HDL cholesterol ratio, were associated with higher RWT in type 2 diabetes patients, only in univariate analysis. The differential findings probably reflect differences in prevalence of obesity and degree of myocardial fat storage between the two populations.^29^

In the LIFE study, concentric remodelling was associated with a three and eight times increased risk of stroke and cardiovascular death after 4.8 years of follow up, respectively.^30^ So, in a way, our findings may be explaining the link between the increased prevalence of congestive heart failure and stroke seen among black diabetic patients.^31^

Of note, an independent association between gender and measures of LV geometry was not found in the present study population, partly contrasting with findings in African Americans participating in the Atherosclerosis Risk in Community (ARIC) study, which reported that diabetic women had more concentric LV geometry, but similar prevalence of LV hypertrophy as men.^32^

We have shown that a simple algorithm using every-day clinical and laboratory tests (type of diabetes, hypertension, obesity and albuminuria) may be used to identify three out of four high-risk diabetic patients with increased RWT. This is very important in a setting such as Tanzania where echocardiography is not readily available. Of note, following this algorithm, a patient with type 2 diabetes with any of the other three risk factors, or a type 1 diabetes patient having any two of the other three risk factors will have a 76% chance of having cardiac target-organ damage as well.

## Conclusion

We have shown that abnormal LV geometry was common in this diabetic population. In particular, increased RWT was present in 58% of patients and demonstrated as a marker of subclinical cardiac target-organ damage. Furthermore, using the clinical risk factors, type of diabetes, hypertension, obesity and albuminuria, 76% of diabetic patients with increased RWT can be identified.
